# The Ketogenic Diet Alters the Hypoxic Response and Affects Expression of Proteins Associated with Angiogenesis, Invasive Potential and Vascular Permeability in a Mouse Glioma Model

**DOI:** 10.1371/journal.pone.0130357

**Published:** 2015-06-17

**Authors:** Eric C. Woolf, Kara L. Curley, Qingwei Liu, Gregory H. Turner, Julie A. Charlton, Mark C. Preul, Adrienne C. Scheck

**Affiliations:** 1 Neuro-Oncology Research, Barrow Brain Tumor Research Center, Barrow Neurological Institute dba St. Joseph’s Hospital and Medical Center, Phoenix, Arizona, 85013, United States of America; 2 School of Life Sciences, Arizona State University, Tempe, Arizona, 85281, United States of America; 3 BNI-ASU Center for Preclinical Imaging, Barrow Neurological Institute dba St. Joseph’s Hospital and Medical Center, Phoenix, Arizona, 85013, United States of America; 4 Neurosurgery Research, Barrow Neurological Institute dba St. Joseph's Hospital and Medical Center, Phoenix, Arizona, 85013, United States of America; University of Portsmouth, School of Pharmacy & Biomedical Sciences, UNITED KINGDOM

## Abstract

**Background:**

The successful treatment of malignant gliomas remains a challenge despite the current standard of care, which consists of surgery, radiation and temozolomide. Advances in the survival of brain cancer patients require the design of new therapeutic approaches that take advantage of common phenotypes such as the altered metabolism found in cancer cells. It has therefore been postulated that the high-fat, low-carbohydrate, adequate protein ketogenic diet (KD) may be useful in the treatment of brain tumors. We have demonstrated that the KD enhances survival and potentiates standard therapy in a mouse model of malignant glioma, yet the mechanisms are not fully understood.

**Methods:**

To explore the effects of the KD on various aspects of tumor growth and progression, we used the immunocompetent, syngeneic GL261-Luc2 mouse model of malignant glioma.

**Results:**

Tumors from animals maintained on KD showed reduced expression of the hypoxia marker carbonic anhydrase 9, hypoxia inducible factor 1-alpha, and decreased activation of nuclear factor kappa B. Additionally, tumors from animals maintained on KD had reduced tumor microvasculature and decreased expression of vascular endothelial growth factor receptor 2, matrix metalloproteinase-2 and vimentin. Peritumoral edema was significantly reduced in animals fed the KD and protein analyses showed altered expression of zona occludens-1 and aquaporin-4.

**Conclusions:**

The KD directly or indirectly alters the expression of several proteins involved in malignant progression and may be a useful tool for the treatment of gliomas.

## Introduction

Glioblastoma multiforme (GBM) is the most aggressive type of brain tumor. Despite surgery, radiation and chemotherapy, these patients have an average life expectancy of 12–18 months and less than 10% survive 5 years [1]. Although there have been advances in the development of novel targeted treatments, these therapies face the challenge of overcoming phenotypic variability resulting from tumor heterogeneity. One phenotypic trait shared by virtually all cancer cells is dysregulation of metabolism.

A metabolic shift toward glycolysis regardless of oxygen availability has been observed in GBM and a variety of other cancers. This phenomenon, first described by Otto Warburg and called the Warburg Effect [2], supports the synthesis of biomolecules needed to sustain rapid proliferation, reducing utilization of the tricarboxylic acid cycle and oxidative phosphorylation. This aberrant metabolism found in tumor cells is now considered a hallmark of cancer [3] and a potential therapeutic target [4]. One novel approach to targeting tumor metabolism is through the use of a therapeutic ketogenic diet (KD). The KD is a high-fat, low-carbohydrate diet that has been implemented in the non-pharmacologic treatment of refractory epilepsy. We and others have demonstrated that this diet enhances survival in preclinical models of malignant gliomas [5]. We also found that the KD altered the expression of genes involved in the oxidative stress response and reduced reactive oxygen species (ROS) in tumors [6]. ROS levels are often increased in cancer [7] and they play a role in a variety of pathways including tumor angiogenesis and growth through the regulation of hypoxia inducible factor-1 (HIF-1) and the vascular endothelial growth factor (VEGF) pathway [8]. Further, we demonstrated that radiation in combination with KD was synergistic, and survival was significantly increased over radiation treatment alone [9]. While the preclinical work on the KD has led to a limited number of clinical trials, the mechanisms through which the KD exerts its anti-tumor effects have not been fully elucidated.

Pathways long known to be associated with tumor cell growth, escape from apoptosis, angiogenesis, and therapy resistance have now been linked to cellular metabolism. For example, hypoxia is a fundamental biological phenomena commonly found in GBM. It drives glycolysis, energy metabolism and other malignant processes including angiogenesis and invasion [10]. HIF-1 is the key transcriptional regulator of the hypoxic response, upregulating many critical genes involved with tumor progression. HIF-1 activation facilitates neoangiogenesis and degradation of the extracellular matrix (ECM) by upregulating expression of VEGF, its associated receptors, and matrix metalloproteinases (MMPs). Further, the malignant hallmarks driven by hypoxia and HIF-1 expression have been implicated in radio- and chemo-resistance, leading to poor patient prognosis [11].

The current study explores the KD in the context of tumor hypoxia and angiogenesis. We show for the first time that the KD given *ad libitum* significantly reduces the key modulators of hypoxic response: carbonic anhydrase IX (CA IX) and HIF-1α, and decreases the activation of nuclear factor—kappa B (NF-κB). In addition, we found that the KD reduces the expression of VEGFR2 while decreasing tumor microvasculature and altering the expression of several other proteins that modify the tumor microenvironment during hypoxia. Our findings suggest that the KD affects the expression of key proteins involved with the hypoxic response that drives tumor growth and progression.

## Materials and Methods

### Ethics statement

This study was performed in strict accordance with the recommendations in the Guide for the Care and Use of Laboratory Animals of the National Institutes of Health. The protocol was approved by the Institutional Animal Care and Use Committee of St. Joseph’s Hospital and Medical Center (protocol number 334 (A3510-01)). All surgery was performed under ketamine/xylazine anesthesia, and every effort was made to minimize suffering.

### GL261-luc2 mouse model of glioma

Bioluminescent GL261-luc2 cells were derived and grown as previously described [9]. Cells were harvested and implanted into 10 week old female C57BL/6—cBrd/cBrd/Cr (albino C57BL/6) mice (National Cancer Institute at Frederick Animal Production Program, Frederick, MD) as described [6,9,12].

### Treatment and animal monitoring

Animals were fed standard rodent chow (SD) for 3 days following surgery [12], and were then randomized to remain on SD *ad libitum* or changed to KetoCal (KC; Nutricia North America, Gaithersburg, MD) *ad libitum*. KC is a nutritionally complete diet providing a 4:1 ratio of fats to carbohydrates plus protein (72% fat, 15% protein, and 3% carbohydrate). KC paste (2:1 mix of KC and water) was provided to animals daily. Tumor burden was quantified by bioluminescence as described [6]. Serum β-hydroxybutyrate and glucose levels were measured using a Precision Xtra blood monitoring system (Abbott Laboratories, Abbott Park, IL) and animals were weighed every 3–4 days.

### *In vivo* imaging of hypoxia

At 21 days post-implantation, HypoxiSense680 (2 nmol/100 μl; PerkinElmer, Inc., Waltham, MA) was administered by intraperitoneal injection and animals were imaged 24 hours later using the IVIS Spectrum *in vivo* imaging system (675 ex/720 em;). Spectral unmixing was performed and quantitation was done using the system’s Living Image 4.3 software. Each tumor bearing animal was imaged prior to HypoxiSense680 injection to quantitate signal generated by tissue autofluorescence. Non-tumor bearing mice were injected with HypoxiSense680 and imaged 24 hours later to quantitate non-specific binding of the probe.

### Western blotting

On day 21 post implantation, animals were euthanized, tumor tissue was processed and western blots were performed as previously described [13]. Protein concentrations were determined using the Pierce BCA protein assay kit (Thermo Scientific, Rockford, IL, USA). Primary antibodies included CA IX (1:1000; Proteintech, Chicago, IL), HIF-1α (1:500; Bioss, Woburn, MA), NF-κB (1:1000; Cell Signaling Technology), phosphorylated NF-κB (p-NF-κB; 1:1000; Cell Signaling Technology), CD31 (1:5000; Novus Biologicals, Littleton, CO), VEGF (1:1000; Santa Cruz Biotechnology, Dallas, TX), VEGF receptor 2 (VEGFR2; 1:5000; Novus Biologicals), MMP-2 (1:500; Bioss), MMP-9 (1:600; Novus Biologicals), Vimentin (1:200, Santa Cruz Biotechnology), zona occudins-1 (ZO-1; 1:200; Biorbyt, Cambridge, UK), Occludin (1:40; Biorbyt), Aquaporin-1 (1:1000; Abcam, Cambridge, UK) and Aquaporin-4 (1:1000; Abcam), and β-actin (1:6000; Abcam). Densitometry was used to determine the ratio of the target to β-actin (VisionWorks LS 7.1 software; UVP, Upland, CA).

### Immunohistochemistry

Formalin fixed paraffin-embedded tissue sections were deparaffinized followed by antigen retrieval in sodium citrate buffer (10 mM; pH 6.0) at 98°C for 25 min. Sections were blocked with 1% bovine serum albumin in TBST for 1 hour at room temperature (RT) and incubated in Image-iT FX signal enhancer (Life Technologies, Carlsbad, CA) for 30 minutes at RT. Sections were incubated with anti-CD31 antibody (1:2500, Novus Biologicals) overnight at 4°C followed by AlexaFluor 488 goat anti-rabbit IgG antibody (1:1500, Life Technologies) for 1 hour at RT. Lipofucsin induced autofluorescence was reduced using 1 mM CuSO_4_ diluted in 50 nM ammonium acetate buffer (pH 5) as previously described [14]. Sections were counterstained with VectaShield mounting media containing DAPI (Vector Laboratories, Burlingame, CA) and imaged using a Zeiss LSM 710 microscope (Carl Zeiss International, Gottingen, Germany) and Zen software (Zeiss). Total CD31 staining was determined by averaging the pixel density in 5 random, 200x fields within the same tumor for each animal using Image J software (NIH, Bethesda, MD). The percentage of CD31 positive staining per analyzed area of the tumor was determined by normalized pixel threshold analysis.

### Gene expression analysis

Total cellular RNA from tumor tissue was isolated using TRIzol LS Reagent (Life Technologies) and conditions specified by the manufacturer. RNA was further purified using an RNeasy Mini Kit (Qiagen, Valencia, CA), DNased using TURBO DNase (Ambion, Life Technologies) and the quality was determined using an Agilent 2100 Bioanalyzer (Agilent Technologies, Palo Alto, CA). The Mouse Cancer RT^2^ Profiler PCR Array was performed as a fee-for-service by Qiagen.

### Measurement of peritumoral edema

On Day 14 following tumor implantation, MR images were acquired on a Bruker Biospec 7.0T small animal MR scanner (Bruker Medizintechnik, Karlsruhe, Germany) with 72mm transmit coil and a surface receive coil. A multiple slice 2D T2-weighted RARE sequence was acquired as reference image (29 slices, 0.1 mm x 0.1 mm, thickness 0.5 mm, TR = 4000 ms, effective TE = 60 ms, RARE factor = 8) to locate the slice with maximum tumor size. A multi-echo T2 relaxometry sequence was used for T2 mapping, in which a series of T2-weighted images were obtained at 28 different echo times, starting from 10.57ms with 10.57ms increments, with in-plane resolution of 0.078 mm x 0.078 mm, slice thickness 1.0 mm, matrix size = 192 x 192, field of view = 15 mm x15 mm, repetition time = 3000 ms. The T2 map was derived by single-exponential fitting of the data.

### Statistical methods

Statistical analyses were performed using GraphPad Prism v 5.04 (GraphPad Software, San Diego, CA). All values are represented as the mean ± SD and significance was determined using the Student’s *t* test. P < 0.05 was considered statistically significant.

## Results

### Animals fed KC show increased β-hydroxybutyrate levels and a reduction in blood glucose

**Fig 1:**
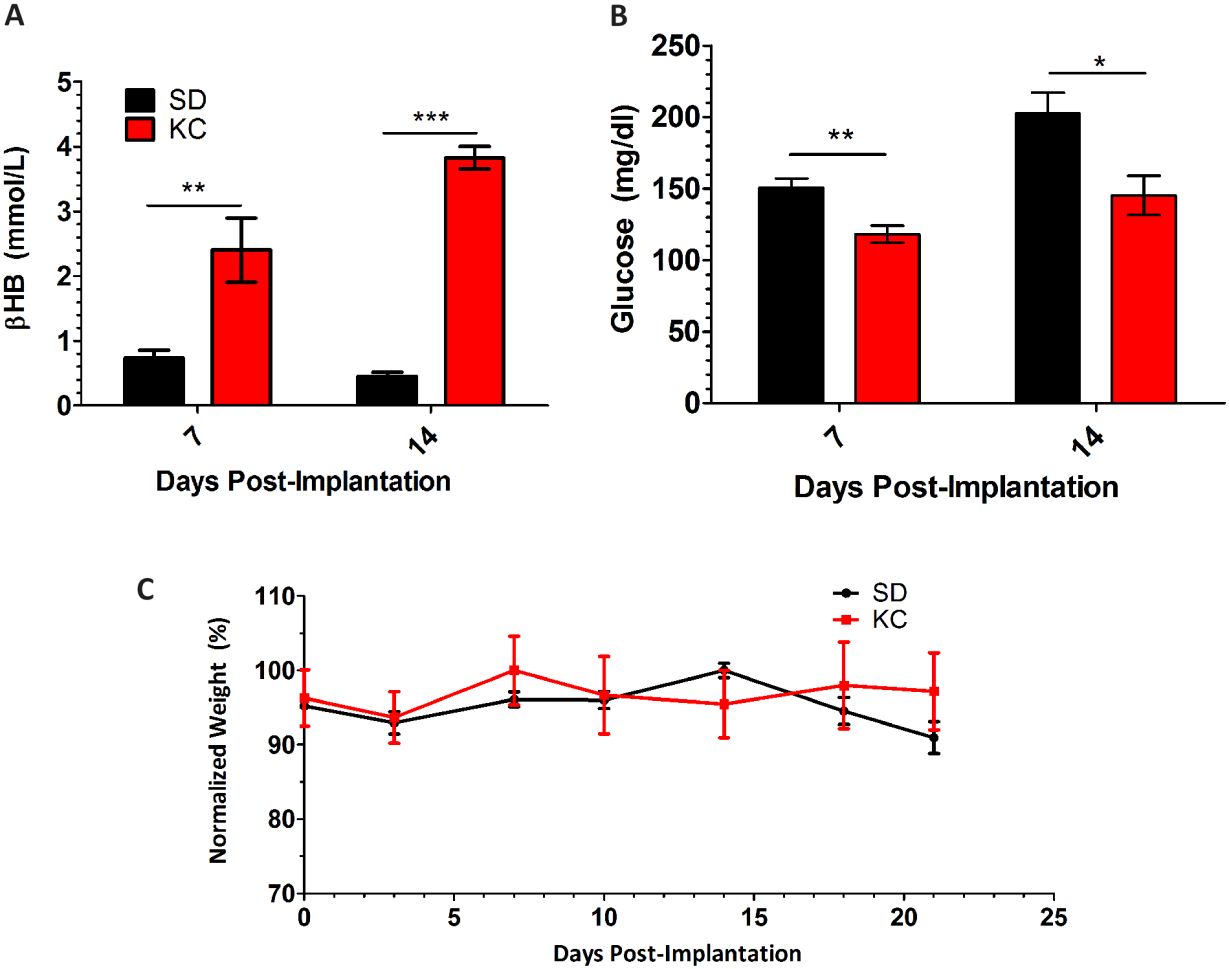
Weight, βHB and glucose measurements. Blood ketone and glucose measurements show (A) higher βHB and (B) lower glucose in KC treated animals (C) weight measurements were taken every 3 days. Graph shows weights normalized to the average starting weight of each group. (N = 5; *p < 0.05; **p < 0.01; ***p < 0.001).

Animals fed KC had a statistically significant increase in blood βHB levels (Fig 1A) and decreased blood glucose (Fig 1B) both 7 and 14 days post-implantation. Body weight remained relatively stable throughout the course of the experiment (Fig 1C). Body weights demonstrated a slight decline throughout the experiment as the tumor grows and animals start becoming symptomatic, consistent with our previous results [9]. At day 14 post-implantation there was a slight decrease in average body weight for the animals fed KC however this was not significant. On day 21 post-implantation, animals were euthanized to obtain tissue for *ex vivo* analyses.

### KC reduces *in vivo* expression of carbonic anhydrase IX

**Fig 2:**
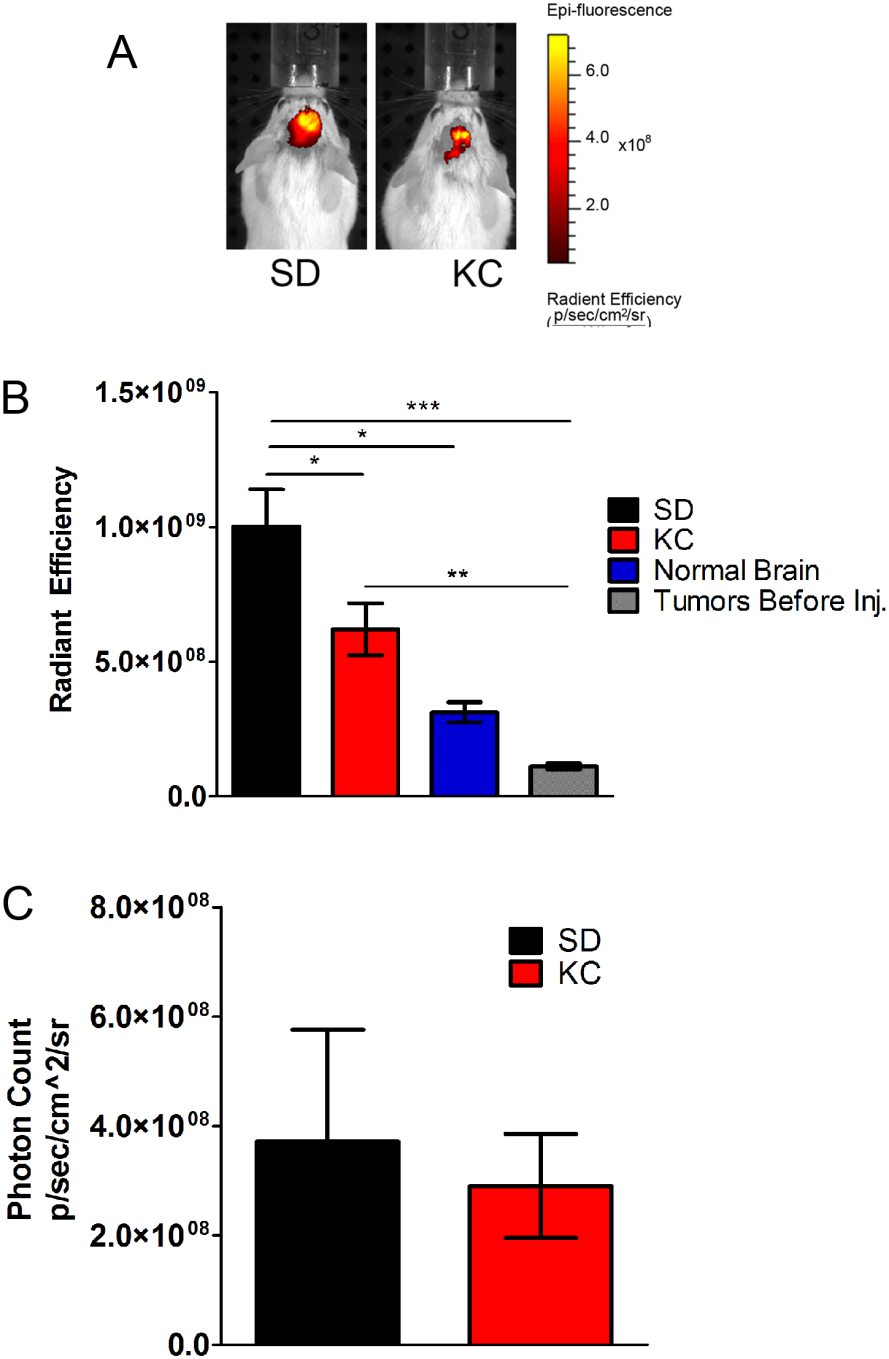
*In vivo* imaging of hypoxia. (A) The fluorescent probe HypoxiSense 680 was used to analyze hypoxia *in vivo* 21 days following tumor implantation. (B) Fluorescent signal was quantitated from tumor bearing mice (N = 5; *p < 0.05). Animals were imaged prior to injection to analyze tissue autofluorescence (“Before injection”; N = 5; ***p < 0.001). Non-tumor bearing mice were injected to analyze non-specific binding (“Normal Brain”; N = 2; *p < 0.05). (C) Tumor bioluminescence imaging showed no significant difference between SD and KC (N = 5).

To determine the effect of the KC on hypoxia, the fluorescent imaging agent HypoxiSense 680 was used to detect *in vivo* expression of the hypoxia marker carbonic anhydrase IX (CA IX) on the surface of tumor cells. Animals maintained on KC had a statistically significant reduction in HypoxiSense 680 signal when compared to animals fed SD (Fig 2A and 2B). Tumor bearing animals were imaged prior to injection of the probe to quantitate signal generated by tissue autofluorescence (Fig 2B). Non-tumor bearing mice were injected with the probe and imaged 24 hours later to quantitate non-specific binding of the probe (Fig 2B). To confirm that this effect was not a result of tumor size, bioluminescence was measured and showed no significant difference between treatment groups (Fig 2C).

### KC reduces expression of proteins involved with the hypoxic response

**Fig 3:**
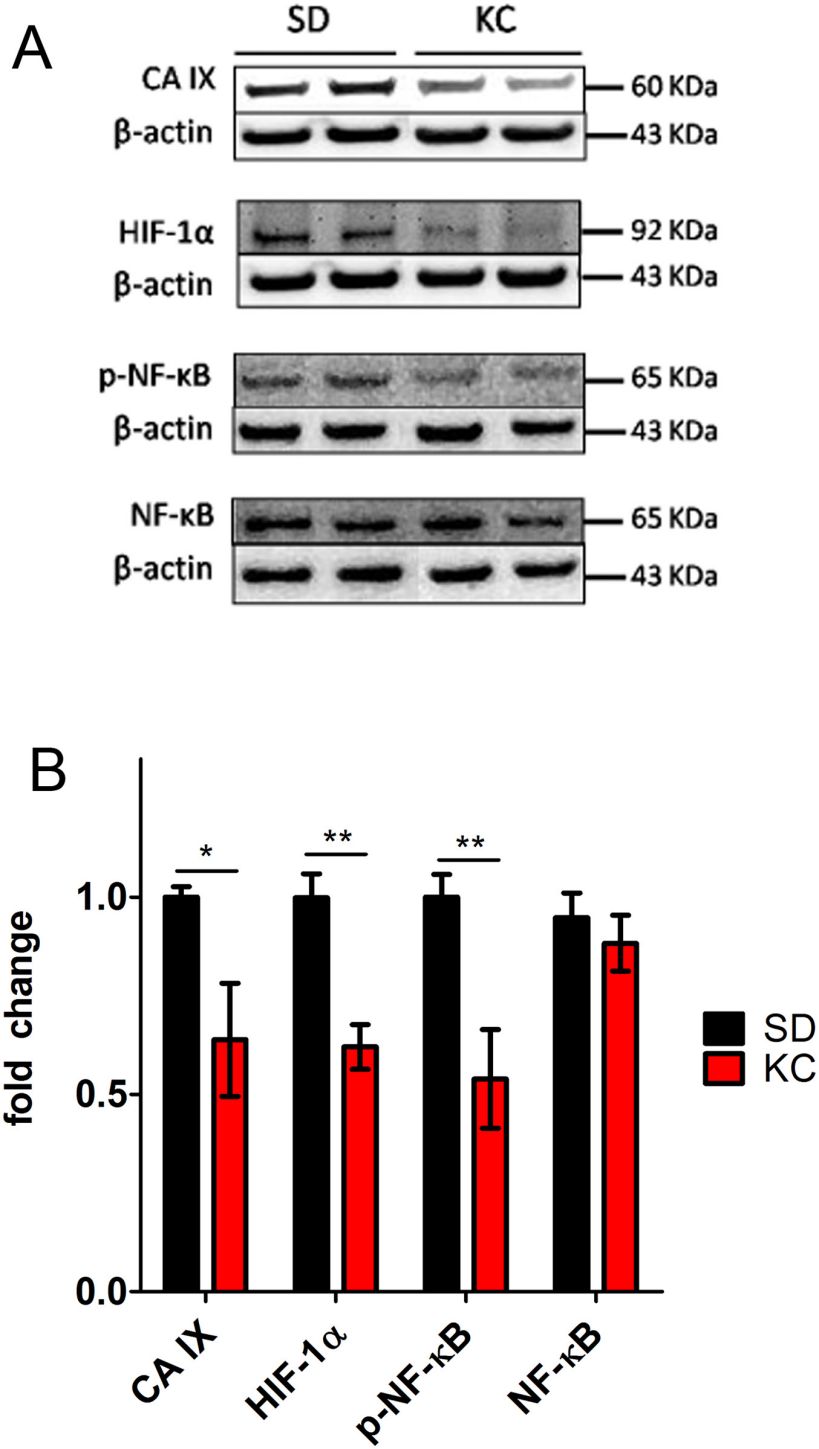
Western blot analysis of CA IX, HIF-1α, phospho-NF-κB, and total NF-κB. (A) Western blots showing two representative samples per treatment group. (B) On day 21 post-implantation expression was quantified and represented as a fold change from SD (N = 6; *p < 0.05; **p < 0.01).

Western blot analysis of tumor lysates was performed to study the effects of KC on the expression of proteins important in the hypoxic response. Both CA IX and HIF-1α levels were significantly reduced in the tumors from animals fed KC when compared to those fed SD (Fig 3; N = 6; *p < 0.05; **p < 0.01). Further, although there was no difference in total NF-κB expression, there was a significant reduction in the phosphorylated form, suggesting a reduction in the activation of NF-κB (Fig 3).

### KC reduces tumor microvasculature

**Fig 4:**
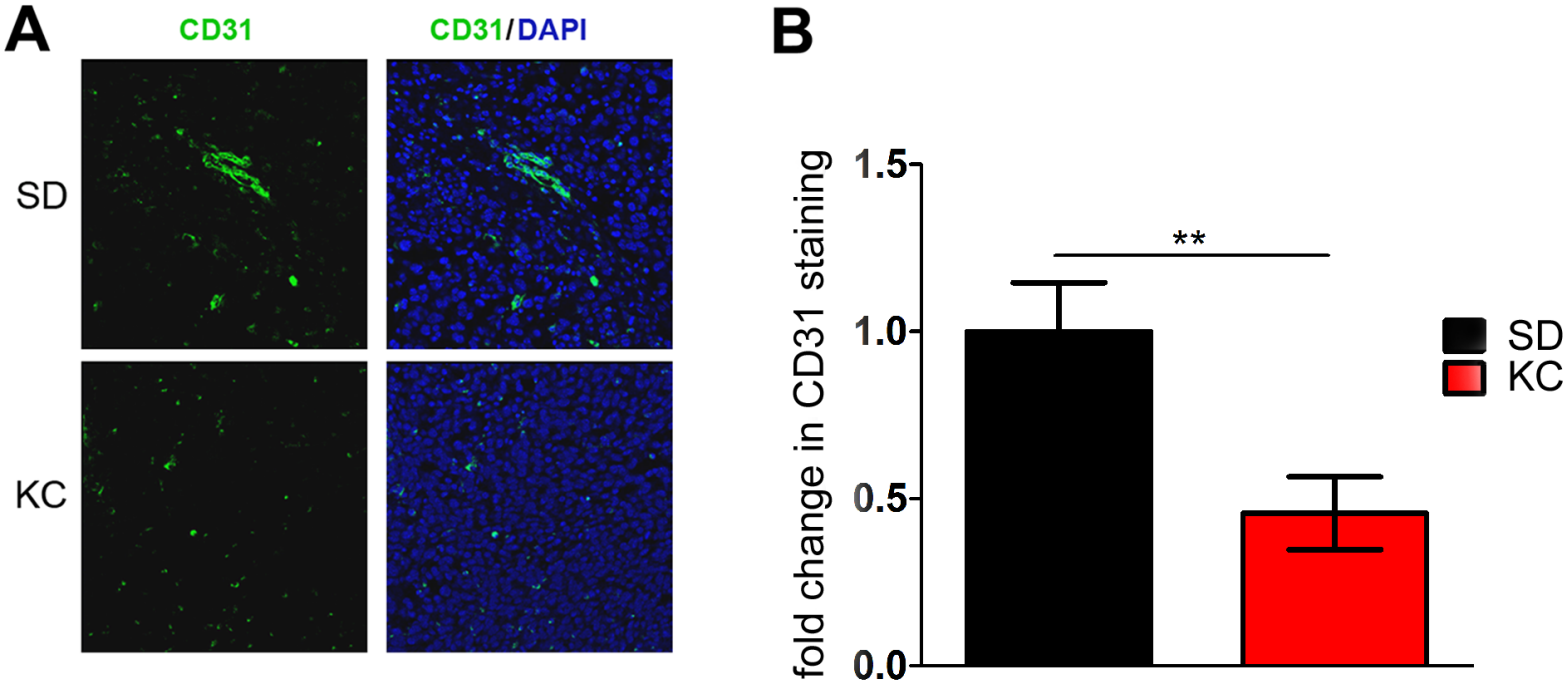
Analysis of tumor microvasculature components and gene expression. (A) CD31 immunostaining of tissue harvested at 21 days post-implantation. Representative images are shown. (B) Quantification of CD31 staining was performed on 2 independent tumors from each group. Data calculated as the average pixel density in 5 random, 200x fields within the same tumor and represented as a fold change from SD (**p < 0.01).

**Fig 5:**
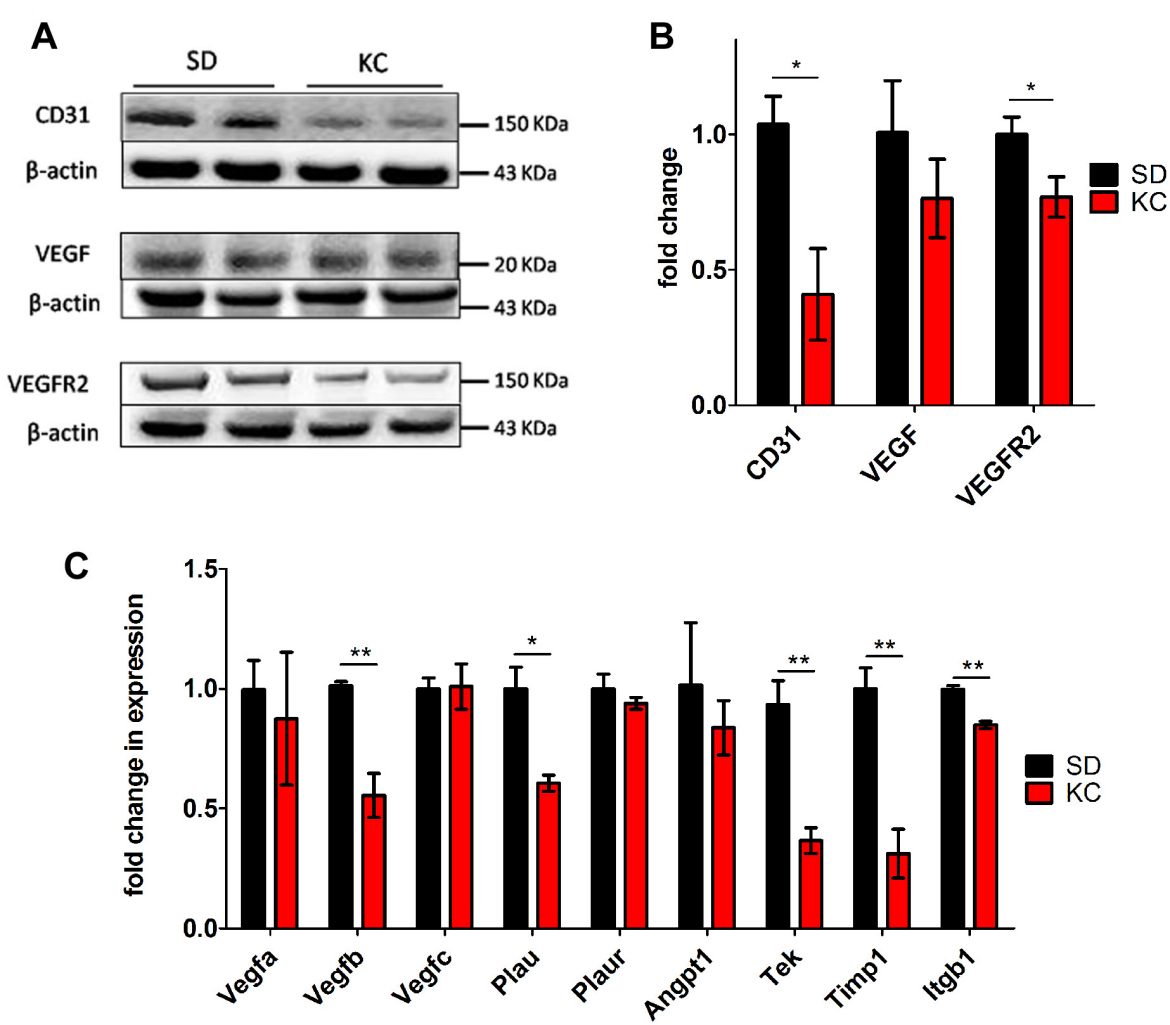
Western blot analysis of CD31, VEGF and VEGFR2. (A) Western blots showing two representative samples per treatment group. (B) At day 21 post implantation expression was quantified and represented as a fold change from SD (N = 6 for SD and N = 5 for KC; *p < 0.05). (C) Expression of genes involved in angiogenesis was analyzed using RT-PCR. Data represented as the fold change difference in expression from SD tumors (N = 3; *p < 0.05; **p < 0.01).

To assess the effect of KC on tumor microvasculature and the expression of the angiogenic marker CD31, we performed immunohistochemical staining of paraffin-embedded tissue sections (Fig 4A). Quantitation of staining demonstrated a statistically significant decrease in the percentage of CD31 positive areas within the tumors of animals fed KC (Fig 4B). These results were confirmed with western blot analysis of CD31 from whole lysate of GL261-luc2 tumors (Fig 5A) which demonstrated a statistically significant 2-fold decrease in expression of CD31 in animals fed KC when compared to SD fed animals (Fig 5B).

### KC alters expression of genes involved in angiogenesis and vascular modeling

The expression of key regulators of tumor angiogenesis, VEGF and VEGFR2, was analyzed by western blot. There was no difference in total VEGF expression; however, a significant reduction in VEGFR2 expression was found (Figs 5A and 4B).

Tumor RNA was analyzed from animals fed KC and animals fed SD using a mouse Cancer RT^2^ Profiler PCR Array (Qiagen). The expression of a number of genes involved in various angiogenenic processes were found to be altered by KC (Fig 5C). Of those genes, *Vegfb*, *Plau*, *Timp1*, *Tek*, and *Itgb1* were expressed significantly lower in the tumors from animals fed a KC when compared to tumors from animals fed SD.

### KC alters the expression of genes involved in invasive potential

**Fig 6:**
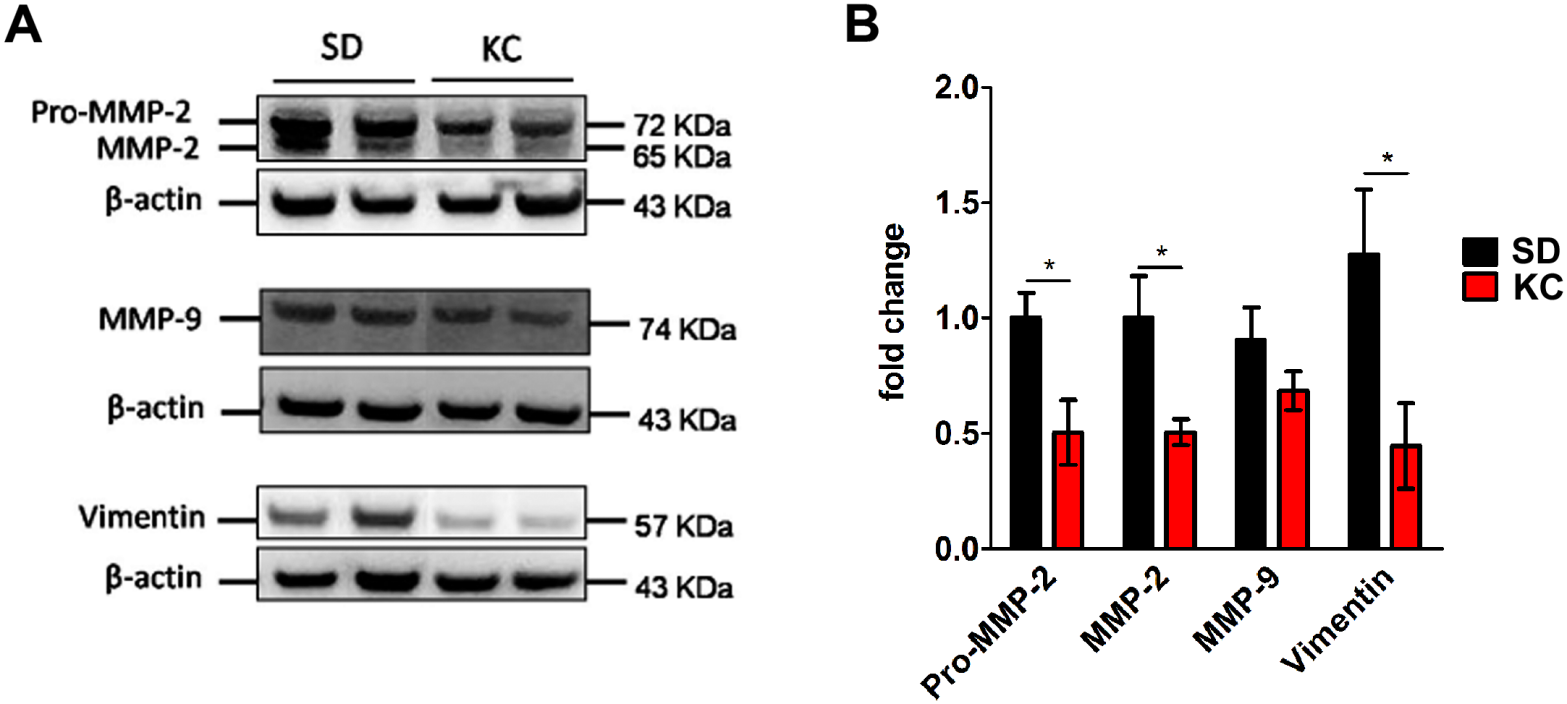
Western blot analysis of pro- and activated- MMP-2, MMP-9, and vimentin. (A) Western blots showing two representative samples per treatment group. (B) On day 21 post-implantation expression was quantified and represented as a fold change from SD (N = 6 for SD; N = 5 for KC; *p < 0.05).

We analyzed tumor invasive potential by performing western blot analysis on tumor tissue to determine the expression levels of vimentin, MMP-9 and both the pro-form and the proteolytically processed activated form of MMP-2. Tumors from animals maintained on KC showed a significant reduction in both the pro- (72 KDa) and activated (65 KDa) form of MMP-2 as well as a reduction in expression of vimentin (Fig 6A and 6B). Although there was a slight reduction in expression of MMP-9 in the tumors from animals maintained on KC, the difference was not statistically significant.

### KC reduces peritumoral edema

**Fig 7:**
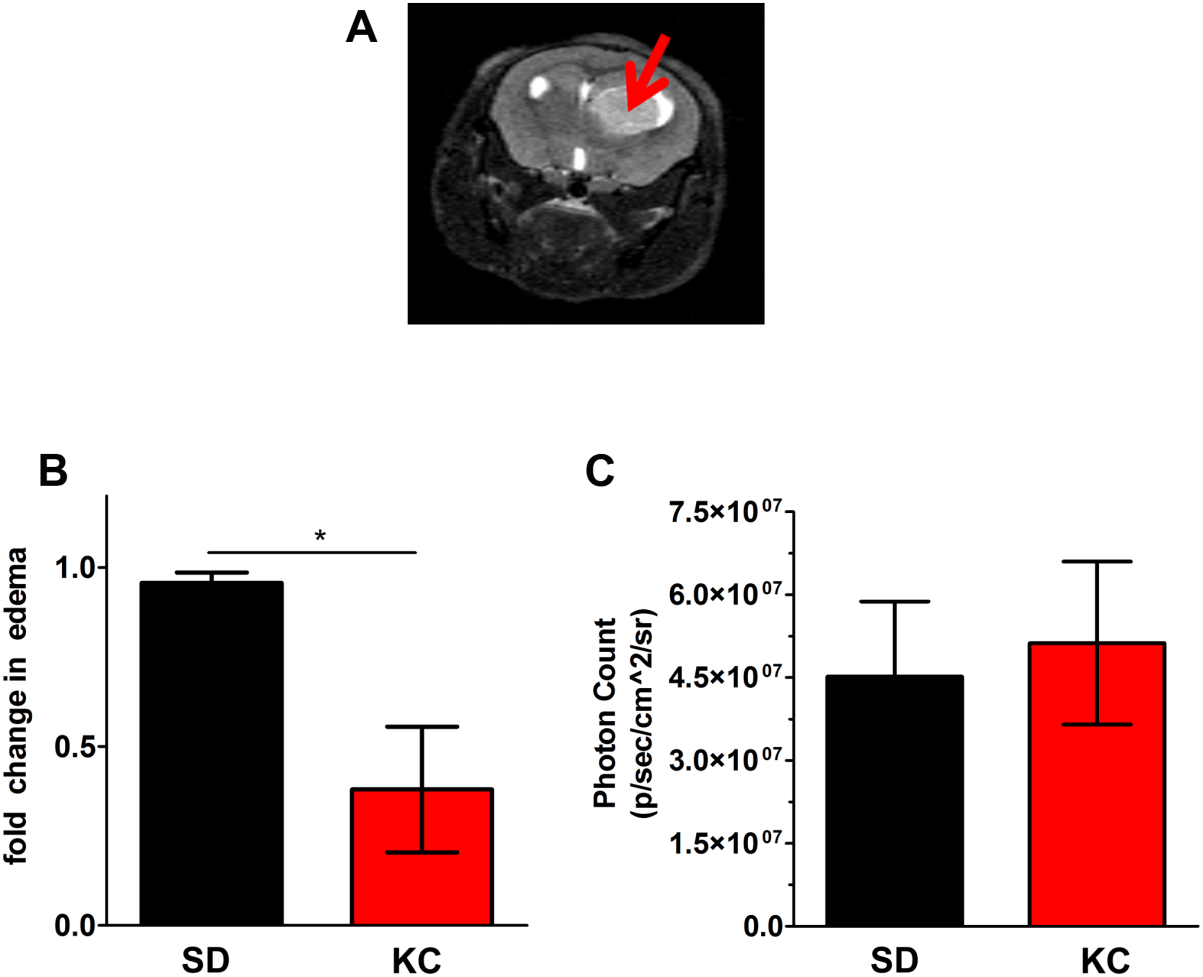
Peritumoral edema measurements. (A) Representative MRI image of tumor bearing mouse (B) 14 days following tumor implantation, edema was measured via MRI. A 2-fold decrease in peritumoral edema was seen in animals fed KC compared to SD (N = 3; *p < 0.05). (C) Tumor bioluminescence imaging showed no significant difference in tumor size between treatments.

The reduction in angiogenesis suggested that animals maintained on KC may have a reduction in peritumoral edema. MRI analysis on day 14 following implantation was done to measure edema (Fig 7A), which is reflected by an increase in T2. Results show a statistically significant 2-fold decrease in peritumoral edema in animals fed KC when compared to those fed SD (Fig 7B). To demonstrate that the reduction in edema with the KC was not purely a function tumor size, bioluminescent tumor signals from the same day were analyzed and there was no statistical difference in signal between groups (Fig 7C).

### KC influences expression of tight junction proteins and aquaporins

**Fig 8:**
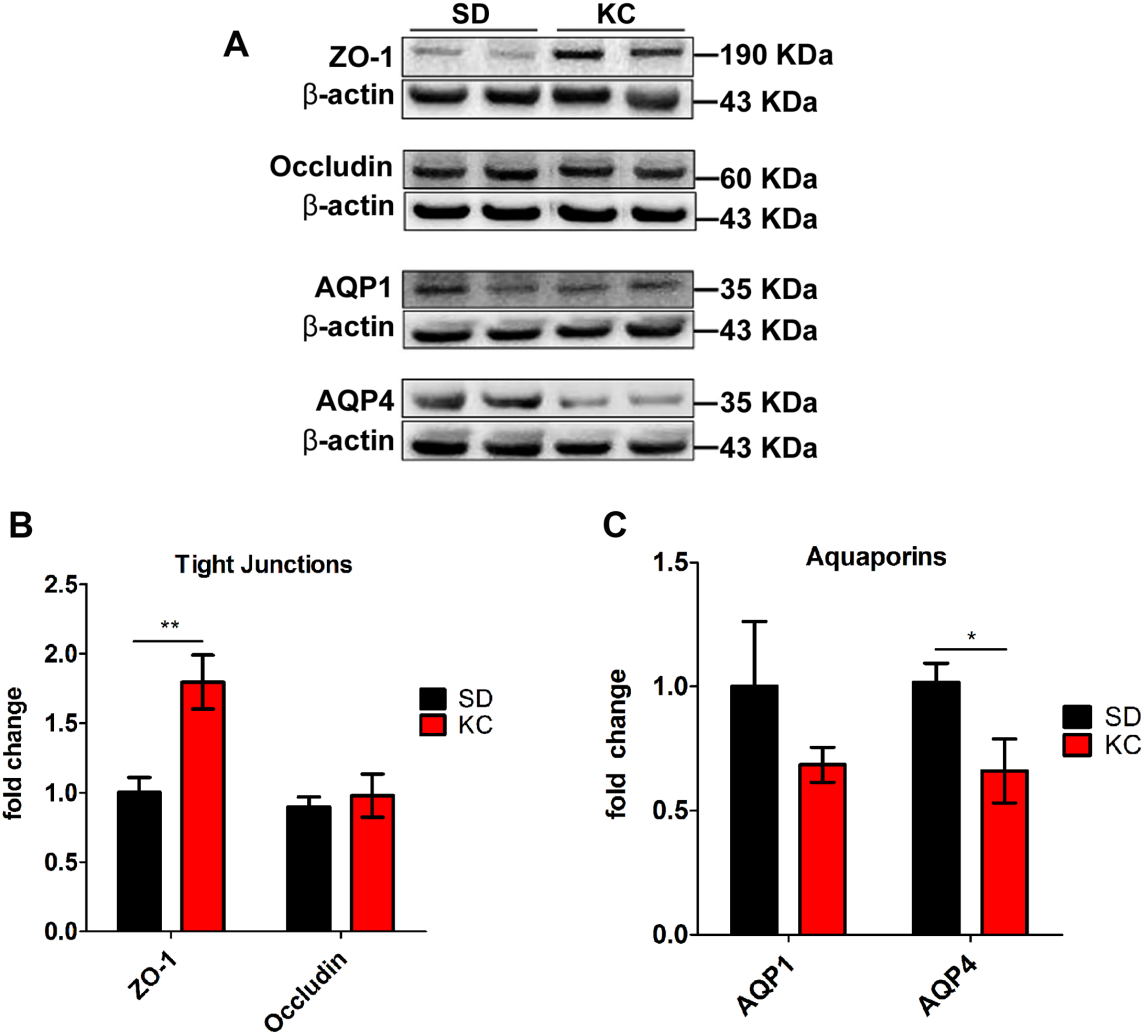
Western blot analysis of ZO-1, occludin, aquaporin-1 and Aquaporin-4. (A) Western blots showing two representative samples per treatment group. (B,C) On day 21 post-implantation expression was quantified and represented as a fold change from SD (N = 6 for SD; N = 5 for KC; *p < 0.05; **p < 0.01).

Western blot analysis of tumor tissue was performed to analyze the expression of proteins involved with peritumoral edema and glioma progression including the tight junction proteins, ZO-1 and occludin, as well as the water channeling proteins, AQPN-1, AQPN-4. Tumors from animals fed KC had a significant increase in the expression of ZO-1 but no difference in occludin expression (Fig 8A and 8B). Animals maintained on KC also showed a reduction in expression of AQPN-4 expression but not AQPN-1 (Fig 8A and 8C).

## Discussion

Hypoxia is a fundamental biological phenomena found in a variety of solid tumors. HIF-1 activity is regulated by rapid stabilization of the HIF1α subunit and acts as a crucial transcriptional factor in the cellular response to hypoxia [15]. HIF-1 can facilitate angiogenesis and invasion through upregulation of various target genes including VEGF, VEGF receptors and MMPs [15]. CA IX expression is also controlled by HIF-1α and is thus considered a marker for hypoxia. Overexpression of CA IX is common in malignant glioma and correlates with poor patient survival [16]. The current study found a reduction in the expression of HIF-1α and CA IX in tumors from animals maintained on the KD, suggesting reduced hypoxia in these tumors. We propose that the resulting alterations in angiogenesis, specifically involving the VEGF pathway, may be a factor in the anti-tumor effect seen with the KD. Preclinical and clinical studies have demonstrated that tumor growth and progression can be limited by targeting angiogenesis [17].

In this study we demonstrated that the KD significantly reduced protein expression of VEGFR2, the main receptor responsible for modulating tumor angiogenesis [18]. Recent studies have shown that selective inhibition of VEGFR2 tyrosine kinase induces a radiologic response, normalizes tumor vasculature, and reduces edema while increasing patient quality of life [17]. Further, it has been suggested that a VEGFR2 blockade creates a "normalization window" where vessel structure is normalized and hypoxia is reduced, enhancing radiation therapy in human glioblastoma xenograft models [19]. In our studies, total VEGF at the protein level was not significantly altered by the KD however RNA analysis showed a significant decrease in the expression of *Vegfb*. Although the role of VEGFB in tumor angiogenesis is not well understood, it may be important for angiogenic processes in a context dependent manner [20]. Decreased VEGFB expression is associated with improved survival and may be an indicator of response to anti-angiogenic therapies in ovarian cancer patients [21]. Bevacizumab, the only FDA-approved anti-angiogenesis drug for use in GBM, is a humanized monoclonal antibody that targets VEGFA [22]. While it is has been used for recurrent tumor, it often results in adverse side effects and there is considerable controversy as to its efficacy [23]. Bevacizumab can lead to increased regions of hypoxia, a more invasive, treatment-resistant glioma phenotype and enhanced MMP-2 activation [24]. MMPs remodel the extracellular matrix and alter surface protein expression. Increased expression and activation of MMP-2, which is linked to HIF-1α expression, can lead to cellular invasion and increased blood-brain barrier (BBB) permeability [25]. Silencing pro-MMP-2 resulted in decreased expression of VEGFR2 and enhanced radiosensitivity in a human xenograft model [26]. Protein analysis of tumors from animals fed the KD showed reduced expression of both pro- and activated-MMP-2, and the mesenchymal marker, vimentin, which is upregulated in the epithelial-to-mesenchymal transition (EMT) that occurs within invasive glioma cells [27,28]. Together these results suggest that a KD may reduce the invasive potential of cells within the tumor. If metabolic therapy could be used to mimic the beneficial effects of bevacizumab it may provide a less toxic way to limit angiogenesis while limiting pro-invasive selection.

An additional reason angiogenesis presents a clinical challenge is that in contrast to healthy vessels, tumor vasculature is often immature, highly permeable, structurally and functionally abnormal [29]. This can impair delivery of therapeutic agents, alter the tumor microenvironment, drive tumor cell extravasation and allow for the rapid influx of inflammatory cells [30]. Inflammation can lead to peritumoral edema, which is a frequent cause of morbidity and mortality in patients with gliomas. Dexamethasone is the current treatment of choice for peritumoral inflammation and edema, yet it comes with adverse side effects such as hyperglycemia, cardiovascular effects, osteoporosis, weight gain, insomnia, increased susceptibility to infection and cognitive effects which ultimately reduce the quality of life for patients [31]. Hyperglycemia induced by corticosteroids has been linked to shorter survival and may have a negative impact on adjuvant chemoradiotherapy in GBM patients [32–34].

The current study found a reduction in glucose and peritumoral edema in animals maintained on the KD when compared to those fed SD. Animals maintained on the KD also showed a significant reduction in activation of NF-κB in their tumors, which is another key regulator of the transcriptional response to hypoxia [35]. NF-κB is linked to various signal transduction pathways and to transcriptional activation events that mediate inflammation, cell proliferation, cell migration, and angiogenesis [36].

Leaky vasculature and disruption of the blood brain barrier caused by tumors is thought to occur in part because of defects in interendothelial tight junctions. ZO-1 and occludin are critical for maintaining the stability and functions of the tight junctions, and their loss is associated with increased blood brain barrier permeability [37]. ZO-1 expression was significantly increased in the tumors from animals fed the KD when compared to SD; however, there was no difference in expression of occludin. Further, both irradiation and hypoxic conditions have been implicated in the breakdown of the BBB via down-regulation of ZO-1 expression in gliomas [38]. Our results suggest that the KD may help mitigate peritumoral edema and BBB-breakdown by preserving ZO-1 expression.

Brain edema and BBB permeability are also propagated by HIF-1α expression which can increase expression of MMPs and aquaporin-4 (AQP4) [39]. Several groups have proposed the involvement of aquaporins in the pathophysiology of brain edema [40]. Aquaporins are the principle pathway for water movement across most cellular membranes. Of these, AQP1 and AQP4 proteins are found highly expressed in the most malignant gliomas [41]. The current study found a reduction in expression of AQP4 but not AQP1 in the tumors from animals fed a KD. In addition to its well-known function in brain edema, AQP4 has also been recently reported to play a role in cell migration, invasion and survival [40]. Further analysis of gene expression showed tumors in mice fed KC also had significantly lower expression of plasminogen activator urokinase (*Plau*) and angiopoeitin-1 receptor (*Tek*). Both are thought to play a central role in tumor invasion, metastasis and angiogenesis [42]. PLAU inhibitors are being actively explored as anticancer strategies in glioblastomas and may have antiangiogenic properties [43,44]. TEK was also shown to be a key molecular regulator of pathological vascularization in a number of preclinical brain tumor models [45–47]. We found that beta 1 integrin (*Itgb1*) and tissue inhibitor of metalloproteinase 1 (*Timp1*) expression was significantly reduced when animals were maintained on KC. Beta1 integrin has been implicated in tumorigenesis, therapy resistance, invasion and metastasis [48]. Inhibitors of *Itgb1*are being explored as a treatment for various cancers and have been shown to potentiate antiangiogenic therapy in bevacizumab resistant glioblastoma [49]. In addition, low expression levels of TIMP-1 have been associated with longer survival times in GBM patients [50]. Recently TIMP-1 upregulation has also been shown to be involved in mechanisms of developed resistance to anti-VEGF treatment [51]. These results taken together suggest a potential utility of the KD as an adjuvant treatment for brain tumors and highlight the importance of metabolism in tumor progression.

The use of metabolism as a therapeutic target is not new. In 1914, Payton Rous first suggested that restricted food intake reduced tumor growth by reducing the tumor blood supply [52], and early attempts to use metabolism as a therapeutic target often focused on caloric restriction (CR). The combination of CR and the KD (restricted ketogenic diet; RKD) is also being explored as a cancer therapy. Both mouse and human xenograft models have been used to substantiate the anti-proliferative and anti-angiogenic effects of CR and the RKD [53]. Evidence also suggests that CR and the RKD have similar anti-tumor mechanisms to the unrestricted KD Including the alteration of pathways involving with inflammation, normalization of vasculature and peritumoral edema. In a mouse astrocytoma model, CR reduced expression of pro-inflammatory markers, cyclooxygenase-2, NF-κB and macrophage inflammatory protein [13]. Seyfried and colleagues recently used the CT-2A mouse astrocytoma model to show that CR caused a reduction in VEGF expression and promoted vessel maturation, presumably by preventing the association of VEGFR2 and platelet-derived growth factor receptor β [54]. Further, a recent study using CR in a U87 human glioma xenograft model showed a reduction of peritumoral edema and normalization of a variety of factors involved in tumor vessel instability and leakiness, including VEGF expression [55]. While CR and RKD can easily be administered in animal models of malignant tumors and there is anecdotal evidence and a few case reports of efficacy in humans, there has been resistance in the medical community to use CR for cancer patients. An alternative strategy is the KD without caloric restriction as used for the treatment of refractory pediatric epilepsy. This approach may meet less resistance in the clinic, as it has a long safety record and may be easier for patients to maintain [56]. Interestingly, Zhou and colleagues found a trend toward increased survival when administering an unrestricted KD (UKD) in a CT2A mouse astrocytoma model [57], however the results weren’t statistically significant. The authors observed no change in glucose and relatively low βHB levels with the UKD when compared to the data presented above, which may explain the discrepancy. Zhou’s data and the current study highlight the importance of identifying an optimal therapeutic window for βHB and glucose as well as determining which tumor types and subtypes are likely to respond better to the KD.

The mechanisms underlying the anti-tumor benefits of the ketogenic diet, caloric restriction (and intermittent fasting) and other potential metabolic therapies have not yet been fully elucidated; however, preclinical data strongly suggests that metabolic alteration may be a highly effective therapy and may in fact enhance the current standard of care for malignant gliomas. The KD and/or CR are the only therapeutic approaches that simultaneously target multiple hallmarks of cancer such as energy metabolism, inflammation, hypoxia, angiogenesis and invasion. It is not clear whether the KD directly causes all of these changes in the tumor, or if some may be a result of changes in the tumor and/or tumor microenvironment that results from the KD. Indeed, a number of the effects seen such as reduction in peritumoral edema, alterations in hypoxia, etc are known to affect a number of downstream targets. Whether the specific changes are directly or indirectly due to the KD, it is clear that the overall result has powerful anti-tumor effects in our preclinical model of glioma. This not only highlights the interconnectedness of metabolism with other aspects of tumor growth and progression, but suggests a potential utility for the KD in the treatment of malignant brain tumors.
